# The *Wolbachia* strain *w*Au provides highly efficient virus transmission blocking in *Aedes aegypti*

**DOI:** 10.1371/journal.ppat.1006815

**Published:** 2018-01-25

**Authors:** Thomas H. Ant, Christie S. Herd, Vincent Geoghegan, Ary A. Hoffmann, Steven P. Sinkins

**Affiliations:** 1 Centre for Virus Research, University of Glasgow, Glasgow, United Kingdom; 2 Biomedical and Life Sciences, Lancaster University, Lancaster, United Kingdom; 3 Pest and Environmental Adaptation Research Group, Bio21 Institute and the School of BioSciences, The University of Melbourne, Parkville, Victoria, Australia; Stanford University, UNITED STATES

## Abstract

Introduced transinfections of the inherited bacteria *Wolbachia* can inhibit transmission of viruses by *Aedes* mosquitoes, and in *Ae*. *aegypti* are now being deployed for dengue control in a number of countries. Only three *Wolbachia* strains from the large number that exist in nature have to date been introduced and characterized in this species. Here novel *Ae*. *aegypti* transinfections were generated using the *w*AlbA and *w*Au strains. In its native *Ae*. *albopictus*, *w*AlbA is maintained at lower density than the co-infecting *w*AlbB, but following transfer to *Ae*. *aegypti* the relative strain density was reversed, illustrating the strain-specific nature of *Wolbachia*-host co-adaptation in determining density. The *w*Au strain also reached high densities in *Ae*. *aegypti*, and provided highly efficient transmission blocking of dengue and Zika viruses. Both *w*Au and *w*AlbA were less susceptible than *w*Mel to density reduction/incomplete maternal transmission resulting from elevated larval rearing temperatures. Although *w*Au does not induce cytoplasmic incompatibility (CI), it was stably combined with a CI-inducing strain as a superinfection, and this would facilitate its spread into wild populations. *Wolbachia w*Au provides a very promising new option for arbovirus control, particularly for deployment in hot tropical climates.

## Author summary

Mosquito-borne viral diseases represent an increasing threat to human and animal health globally. The mosquito species *Aedes aegypti*, a primary vector of the most significant human arboviral infections including the dengue, Zika and Chikungunya viruses, is highly invasive and is almost ubiquitous in tropical urban areas. Mosquito control remains the main approach for preventing and controlling outbreaks. A novel control strategy that is currently being trialed in several countries utilizes *Ae*. *aegypti* mosquitoes artificially infected with a bacterial symbiont known as *Wolbachia pipientis*. Although many insect species harbor native *Wolbachia* infections, *Ae*. *aegypti* is naturally uninfected. *Wolbachia* lives within host cells and is passed-on from mother to offspring, and can block virus transmission; once released it can invade and persist in host populations. Here we present the infection and assessment of two novel *Wolbachia* strains in *Ae*. *aegypti*. We show that one of the strains, *w*Au, provides particularly strong blocking of dengue and Zika virus transmission and offers greater stability at higher temperatures when compared to *w*Mel—currently the most widely used strain for field releases. These results suggest that *w*Au is promising option for arbovirus control, especially in hot climates.

## Introduction

The mosquito *Aedes aegypti* (Linneaus) is the most important vector of human arboviruses. Although native to Africa it now has a broad distribution throughout the tropics and subtropics and is peridomestic, often laying its eggs in man-made water containers, and displaying a strong preference for feeding on humans. Attempts to reduce the global incidence of dengue fever and stem the spread of recent chikungunya, Zika and yellow fever virus outbreaks have focused on *Ae*. *aegypti* control [1, 2], which has proven challenging. An emerging vector control strategy utilizes mosquitoes artificially transinfected with virus-blocking strains of the alpha-proteobacterium *Wolbachia pipientis* [3]. *Wolbachia* are obligate intracellular endosymbionts naturally found infecting a wide range of terrestrial arthropods. The natural abundance of *Wolbachia* can be partly attributed to its capacity to spread through naïve populations by manipulating host reproduction. Although several forms of reproductive manipulation are found across different arthropod species, the only form observed in mosquitoes is a type of crossing sterility known as cytoplasmic incompatibility (CI). *Wolbachia* modifies the sperm of infected males [4], which results in the generation of non-viable progeny when mated to uninfected females. Infected females, in contrast, ‘rescue’ this sperm modification, producing viable progeny and resulting in a relative fitness advantage that can drive and maintain *Wolbachia* at high population infection frequencies [5].

While *Ae*. *aegypti* is not a natural *Wolbachia* host, stable transinfections with the *w*AlbB strain from *Aedes albopictus* and *w*MelPop/*w*Mel strains from *Drosophila melanogaster* have been generated in the laboratory using embryonic microinjection, with the resulting lines showing reductions in vectorial capacity for a number of arboviruses and other pathogens [6–11]. *Ae*. *aegypti* transinfected with *w*Mel have significantly reduced vector competence for dengue virus [7, 12], yellow fever virus [10], chikungunya [10] and Zika [13] viruses in laboratory challenges. However, mosquito challenges with patient-derived dengue infected blood have indicated that *w*Mel-mediated blocking is incomplete, and modelling predicts that *w*Mel would be insufficient to achieve complete control in some settings [14]. Field trials aimed at spreading *Wolbachia* in *Ae*. *aegypti* for dengue control have to date focused primarily on *w*Mel [15, 16].

Different strains of *Wolbachia* reach varying intracellular densities and display divergent tropism within host tissues; the magnitude of the pathogen inhibition effect shows a positive correlation with *Wolbachia* intracellular density in several species [17–19]. The *w*MelPop strain reaches very high densities in *Ae*. *aegypti*, which probably contributes to an almost complete blocking of dengue virus transmission [6, 12]. However, *w*MelPop imposes significant costs on a variety of traits including reduced longevity, fecundity and egg survival in quiescence [20–23]. These negative fitness effects have made the introduction of *w*MelPop into wild host populations problematic, despite the presence of strong uni-directional CI—recent field trials in Vietnam and Australia failed to achieve population replacement using this strain [24].

Recently several studies have reported the influence of a variety of factors on *Wolbachia* intracellular density. Larval rearing temperature in particular has a significant impact on the densities of *w*Mel and the over-replicating *w*MelPop strain in *Ae*. *aegypti* [25, 26]: exposure of larvae to diurnal rearing temperatures cycling between 27–37°C resulted in dramatic reductions in total *Wolbachia* density, and rates of maternal transmission—ultimately leading to the loss of the *w*Mel and *w*MelPop infections when the high temperature regimes were maintained for more than one generation [26]. In addition to environmental factors, a genetic basis to density determination has been postulated based on duplications of a set of eight genes in the *w*MelPop genome, with copy number reported to correlate with *w*MelPop density in *Drosophila melanogaster* [27]. However, further studies failed to find a straightforward causal role for copy number in *Wolbachia* density regulation [28], but see [29] and [30].

So far, only a few of the vast repertoire of naturally-occurring *Wolbachia* strains have been introduced into *Ae*. *aegypti*. It is important to create and characterize further transinfections in this species since they might provide improved characteristics such as viral blocking under particular environmental conditions, especially in hot climates [3], and offer insights into the regulation of intracellular density and its role in inducing pathogen inhibition and effects on host fitness. Limitations are imposed by the technical demands of embryo cytoplasmic transfer by microinjection and the need for robust lab colonies of the insects to be used as the source of *Wolbachia*. While *w*AlbA and *w*AlbB are naturally found superinfecting *Ae*. *albopictus*, *w*AlbA is maintained at around 10% of the density of *w*AlbB [31] and only *w*AlbB established itself following previous embryo cytoplasm transfers from *Ae*. *albopictus* into *Ae*. *aegypti* [32]. Strain *w*Au does not induce CI in its native host *Drosophila simulans* [33], but confers a notably high degree of protection from pathogenic viruses of *Drosophila* [34, 35]. We therefore aimed to generate and characterize *Ae*. *aegypti* lines containing *w*AlbA and *w*Au for a variety of traits relevant to transmission-blocking and population-replacement potential, in comparison with the previously reported *w*AlbB and *w*Mel transinfections.

## Results

### Strain generation and transmission

Using embryonic cytoplasmic transfer and taking advantage of incomplete maternal inheritance we generated *Wolbachia* transinfected lines carrying strains *w*AlbA, *w*AlbB, *w*Mel, and *w*Au in the same host background of *Ae*. *aegypti*. Each of the *Wolbachia* strains apart from *w*Au was capable of inducing full unidirectional cytoplasmic incompatibility with wild-type mosquitoes, and therefore showed population replacement potential. *w*Au produced no detectable CI (Table 1), consistent with observations in its native host (*Drosophila simulans*) [33] and providing further evidence that it is genetically incapable of generating CI, as opposed to a strain-specific suppression of the phenotype in its native host. *Wolbachia*-infected lines were crossed to determine the crossing types between strains. For lines that induced unidirectional CI with wild-type mosquitoes, no hatching of the resulting eggs was observed, in other words between-strain crosses resulted in complete bidirectional CI.

**Table 1:** Crosses between *Wolbachia*-infected lines.

		Female line				
		*w*AlbA	*w*AlbB	*w*Mel	*w*Au	wt
Male line	*w*AlbA	89.2 (1541)	0 (774)	0 (644)	0 (499)	0 (488)
	*w*AlbB	0 (663)	89.4 (954)	0 (821)	0 (663)	0 (1321)
	*w*Mel	0 (1254)	0 (667)	90.4 (416)	0 (451)	0 (974)
	*w*Au	91.4 (680)	75.3 (237)	92.7 (402)	92.4 (637)	91.4 (810)
	wt	83.7 (771)	79.4 (527)	87.6 (669)	87.6 (296)	87.3 (225)

Eggs are from a single-cage cross of 20 males and 20 females. Females were blood-fed and individualized for oviposition. Numbers show percentage hatch rates with total numbers of eggs counted in parentheses.

Rates of *Wolbachia* maternal inheritance were determined by PCR of progeny from compatible crosses between wild-type males and infected females. All lines showed complete (100%) maternal transmission of all strains in 200 progeny assessed. Since *w*Au does not induce CI, its maintenance in the *w*Au line is facilitated by high rates of maternal inheritance, and it is hypothesized to produce positive host fitness effects under some conditions based on increases in its frequency in native *D*. *simulans* host populations [36]. To assess its stability in *Ae*. *aegypti* populations, 200 individuals from the *w*Au colony were randomly selected and tested for the presence of *Wolbachia* at the fourth, seventh, and tenth generations post initial establishment. Colonies of this line had been maintained at relatively high numbers (>2,000 individuals per generation from G_4_) with no direct selection for *w*Au infection from G_1_ onwards. All individuals tested positive at each generation, indicating that *w*Au is maternally transmitted at very high fidelity under these laboratory conditions.

#### *Wolbachia* intracellular density and tropism

**Fig 1:**
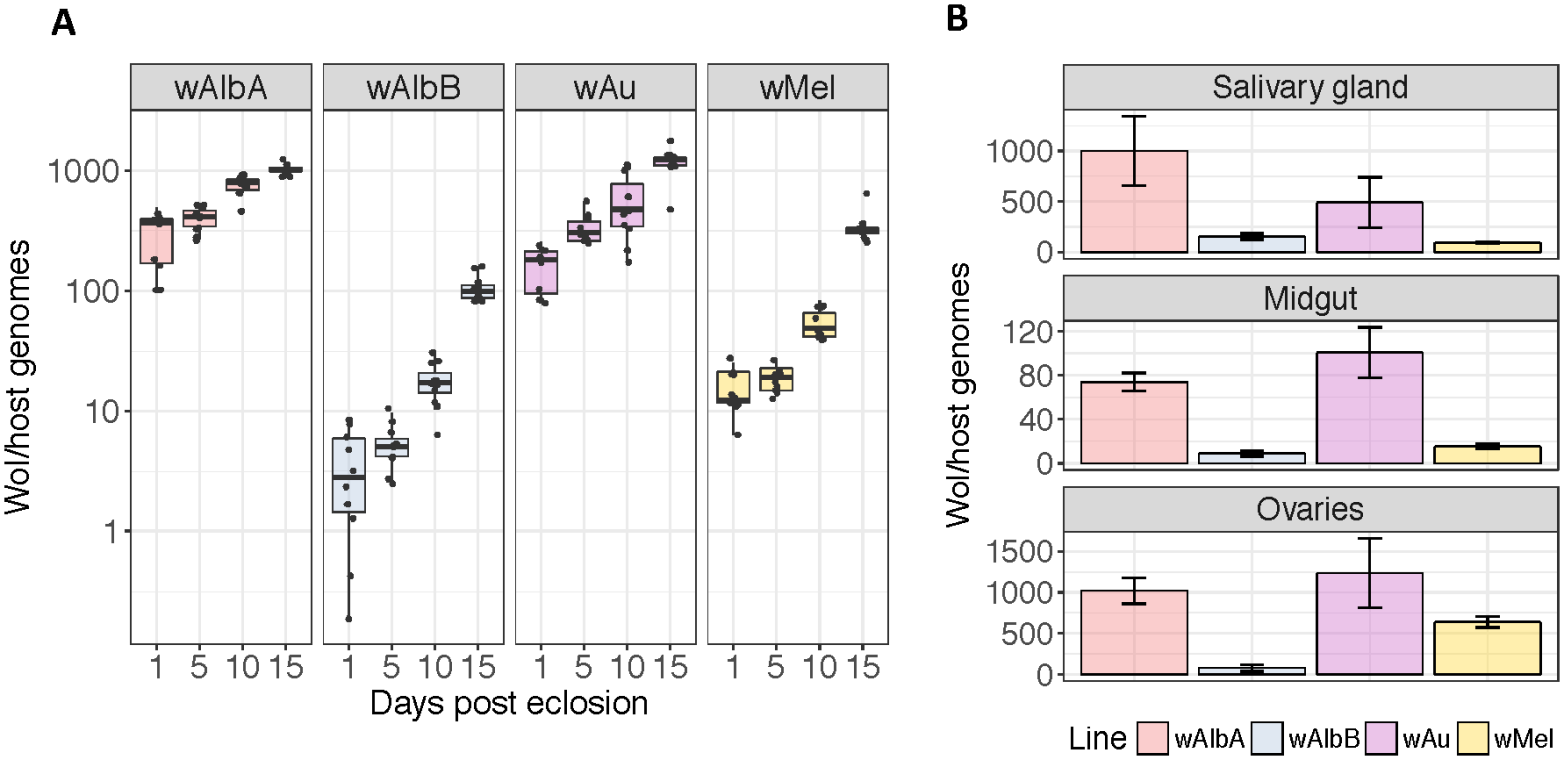
*Wolbachia* densities and tropism in *Aedes* mosquitoes. (**A**) Total *Wolbachia* densities were measured by qPCR in *w*AlbA, *w*AlbB, *w*Au, and *w*Mel carrying *Aedes aegypti* females at varying time points post adult eclosion. Each box represents 10 biological replicates, with pools of 5 females per replicate. The centre of a box plot shows median *Wolbachia* density, edges show upper and lower quartiles, and whiskers indicate upper and lower extremes. (**B**) Total *Wolbachia* densities in dissected tissues measured by qPCR. Each bar represents the average density of 5 biological replicates. For each of the tissue-specific replicates 5 biological replicates of 5 sets of salivary glands, 5 midguts, or 5 ovary pairs were assessed. Error bars show SD. Statistical analyses were performed using a two-tailed Student’s t-test.

Total *Wolbachia* density in each line was monitored over the initial post-transinfection generations by qPCR. Once densities were stable (after five generations for each line) a time-course study was performed to monitor total densities in females over the first 15 days post adult eclosion (Fig 1*A*). Although all lines showed increasing *Wolbachia* density with adult age, there was significant variation in the total densities of the individual *Wolbachia* strains.

Although *w*AlbA reaches only modest densities in its native host *Ae*. *albopictus*, it showed the highest overall density of all the strains assessed at the majority of time-points in *Ae*. *aegypti*. *w*Au also reached comparatively high densities (no significant difference with *w*AlbA at all time points, *p*>0.01, t-test), several fold higher than that of the other native *Drosophila* strain, *w*Mel. *w*AlbB showed the lowest total *Wolbachia* density of the strains at all time points.

It is highly likely that *Wolbachia* tissue distribution plays a key role in determining levels of pathogen inhibition since this phenotype has been reported to be cell-autonomous rather than systemic [6]. Total *Wolbachia* densities were therefore assessed in dissected ovary, midgut and salivary gland tissues (Fig 1*B*). In ovarian tissue *w*Au and *w*AlbA reached similarly high densities (*p* = 0.192, t-test) with *w*AlbB showing the lowest density. In midguts all strains showed relatively low densities compared to the other tissues assessed; in salivary glands *w*AlbA showed the highest density while *w*Mel and *w*AlbB reached relatively low density.

#### Virus inhibition

**Fig 2:**
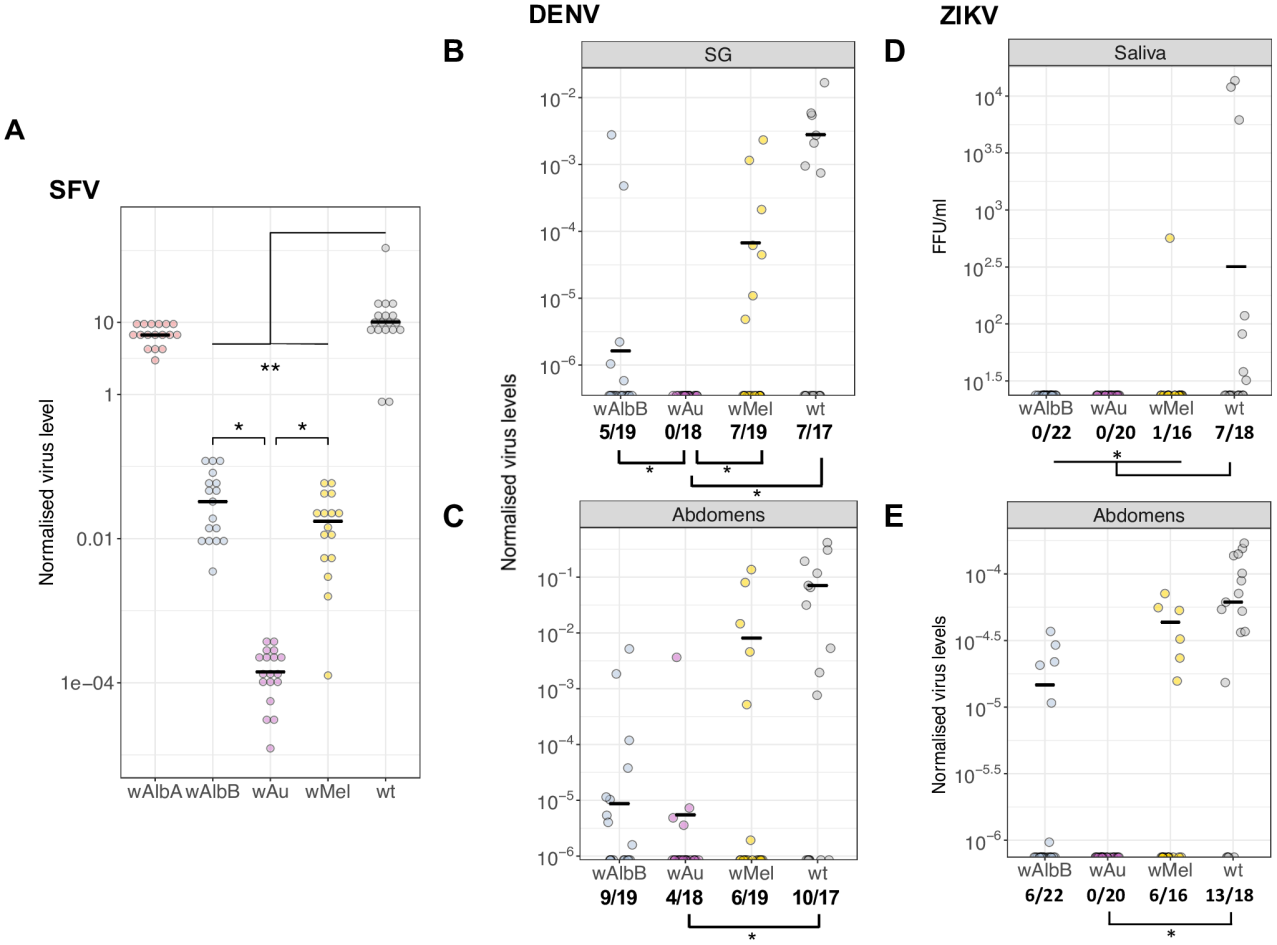
Virus inhibition in *Wolbachia*-infected *Aedes aegypti* lines. (**A**) Semliki Forest virus (SFV) genome copies per host cell following thoracic injection into *Wolbachia*-infected lines and wild-type *Ae*. *aegypti*. Females were left for 10 days prior to total RNA extraction and virus quantification by qPCR. Levels of target RNA sequences were normalized against the RPS17 house-keeping gene. 17, 16, 18, 17 and 17 females were PCR’d for the *w*AlbA, *w*Mel, *w*Au, *w*AlbB and wt, respectively. Statistical analysis was performed using a one-way ANOVA with a Dunnett’s post-hoc test. Dengue-2 (DENV) (**B** and **C**) and Zika (ZIKV) (**D** and **E**) viruses were orally administered to 5-day old females. After an incubation period of 12 days, females were salivated (Zika only) and salivary glands and abdomens dissected. Viral RNA in salivary glands (SG) and abdomens were quantified by reverse-transcriptase qPCR, with viral RNA levels normalized to host RNA using the *RpS17* house-keeping gene. A value of zero for normalized virus levels, indicates no amplification for virus cDNA in that sample. Zika viral titers in saliva were quantified by fluorescent focus assay with results show focus forming units (FFU). Proportions underneath each graph indicate the infection rate for a given strain. Statistical analyses for panels B, C, D and E were performed using a one-tailed Fisher’s exact test comparing rates of virus-positive to virus-negative samples. Black lines indicate median of non-zero values.

To provide an initial indication of the virus blocking potential of the different *Wolbachia* single-infection lines in *Ae*. *aegypti*, the titres of Semliki Forest Virus (SFV), an arbovirus model system, were assessed in whole adult females following intrathoracic microinjection and a ten-day incubation period (Fig 2*A*). *w*Au was more effective in reducing viral load than *w*Mel and *w*AlbB, although all three strains resulted in significantly reduced viral loads compared to wild-type (*p*<0.01, 1-way ANOVA with Dunnett’s). It is notable that the highest density strain (*w*AlbA) was not the most efficient virus blocker, producing no detectable differences in levels of SFV compared with wild-type mosquitoes (*p*>0.3, 1-way ANOVA with Dunnett’s)

To further assess levels of virus transmission blocking, females of the wild-type, *w*Au, *w*Mel and *w*AlbB lines were orally challenged with either a DENV or ZIKV-infected blood meal. Engorged 5-day old females were allowed to incubate virus for 12 days, at which point salivary glands and abdomens were dissected and the presence of viral RNA was quantified by reverse-transcriptase quantitative PCR. Additionally, saliva was collected prior to dissection for the ZIKV infected females, and levels of infectious virus were quantified by fluorescent focus assay (FFA).

For DENV, significant differences in rates of replication and dissemination were observed across the different *Wolbachia* lines. Females of the *w*Au, *w*AlbB, *w*Mel and wild-type lines contained salivary glands positive for DENV RNA, at rates of 0%, 26.3%, 36.8% and 41.2%, respectively (Fig 2*B*). This represents a significant reduction in infection rate of the *w*Au infection compared to wild-type, and for *w*Au compared to the *w*AlbB and *w*Mel lines. In abdomen tissue, infection rates were 22%, 47.4%, 31.6% and 58.8% in the *w*Au, *w*AlbB, *w*Mel and wild-type lines, respectively, and were significantly different between the *w*Au and wild-type lines (Fig 2*C*).

All of the *Wolbachia* infected lines showed significant decreases in ZIKV transmission in saliva compared to wild-type: the *w*AlbB and *w*Au lines completely blocked infectious virus transmission, while 6.3% of the *w*Mel saliva samples were positive, compared to 39% of the wild-type (Fig 2*D*). Similarly, the *w*AlbB and *w*Au females contained no detectable ZIKV in salivary gland tissue, while 12.5% and 50% of the *w*Mel and wild-type were positive, respectively, representing a significant decrease for the *w*Au and *w*AlbB infected lines compared to wild-type (S1 Fig). In abdomen tissue 27.7%, 37.5% and 72.2% were ZIKV positive in the *w*AlbB, *w*Mel and wild-type lines, respectively, while none of the *w*Au abdomens were positive for ZIKV RNA (Fig 2*E*).

#### Effects of high temperature on *Wolbachia* density

Recent reports have shown that exposure of larvae to higher rearing temperatures can significantly affect the density of *Wolbachia* in adults [25, 26], leading to reduced rates of maternal transmission [26]. As *Ae*. *aegypti* larvae are often found in bodies of water experiencing day-time heating, temperature susceptibility could potentially limit the invasive capacity of a *Wolbachia* strain, as well as its ability to inhibit virus transmission. Interestingly, comparisons of the densities of *w*AlbB and *w*Mel in *Ae*. *aegypti* found that the latter was particularly susceptible to cyclical heat treatment, while *w*AlbB was more resilient [26].

**Fig 3:**
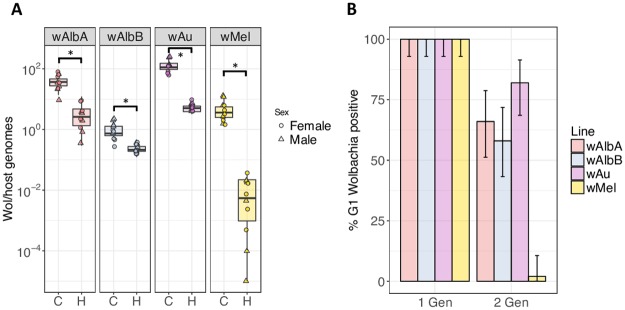
High temperature results in reduced *Wolbachia* densities and maternal leakage. (**A**) Larvae from the *w*AlbA, *w*AlbB, *w*Au, and *w*Mel strains were reared at constant 27°C (C) or with temperature fluctuating between 27–37°C (12hours:12hours) (H) and assessed for *Wolbachia* density by qPCR upon adult emergence. Each point represents a pool of 3 adult mosquitoes. The centre of a box plot shows median *Wolbachia* density, edges show upper and lower quartiles, and whiskers indicate upper and lower extremes. Statistical analyses were performed using a two-tailed Student’s t-test. (**B**) Females reared under larval temperature cycling conditions were allowed to recover upon emergence at a constant 27°C and were crossed to wild-type males with infection rates in resulting progeny assessed (1 Gen). Females reared under heat treatment were mated with wild-type males, and resulting progeny were also reared under high temperature conditions—resulting in two consecutive generations of high temperature treatment. Infection rates were then assessed in the pupae resulting from the second round of larval heating (2 Gen). Error bars show binomial 95% confidence intervals.

The densities and maternal transmission rates of *Wolbachia* in the *w*AlbA, *w*AlbB, *w*Mel and *w*Au lines were examined in adults following exposure of larvae to a rearing temperature regime fluctuating between 27°C and 37°C (12hr:12hr). Consistent with previously published results [25, 26], fluctuating heat regimes during larval rearing resulted in reductions in *Wolbachia* density in emerging adult males and females. Although significant decreases in density were observed for all strains under heat treatment (*p*<0.05 for each comparison, t-test) (Fig 3*A*), the effect was most dramatic for *w*Mel, with a drop in average *Wolbachia* levels in females to 0.017 ± 0.015 (mean ± SD) *Wolbachia*/host cell, an infection density 0.49% that of newly emerged *w*Mel controls raised at a constant 27°C. *w*Au, another native *Drosophila* strain, proved to be more resilient to high temperature treatment than *w*Mel, retaining an infection density of 4.6 ± 1.31 (mean ± SD) *Wolbachia*/host cell in emerging females, 4.47% that of *w*Au controls. The two native mosquito strains tested, *w*AlbA and *w*AlbB, proved to be the most resilient to high temperature fluctuation, retaining infection densities of 4.9 ± 4.4 (mean ± SD) and 0.2 ± 0.06 (mean ± SD) *Wolbachia*/host cell in females, representing 10.72% and 32.12% that of controls, respectively. Strain-specific reductions in *Wolbachia* density were found to be similar in males and females (Fig 3*A*).

Despite the reductions in density, the females in all lines displayed complete transmission to progeny (Fig 3*B*). The effects of two consecutive generations of larval temperature treatment on infection rates was also examined. Females having undergone larval heat treatment were crossed with wild-type males to avoid the effects of CI, and the resulting progeny were also reared under fluctuating high temperature to pupation, at which point infection status was assessed by PCR. We found that each strain contained uninfected individuals, although rates varied widely between *Wolbachia* strains (Fig 3*B*). The *w*Mel line showed an almost complete loss of detectable infection, with only 2% of individuals PCR positive for *Wolbachia*; infection rates were 58%, 66% and 82% for the *w*AlbB, *w*AlbA and *w*Au strains, respectively. These results are consistent with previous findings showing complete loss of *w*Mel and *w*MelPop infections in *Ae*. *aegypti* following two generations of heat cycling [26].

#### *Wolbachia* strains and host fitness

**Fig 4:**
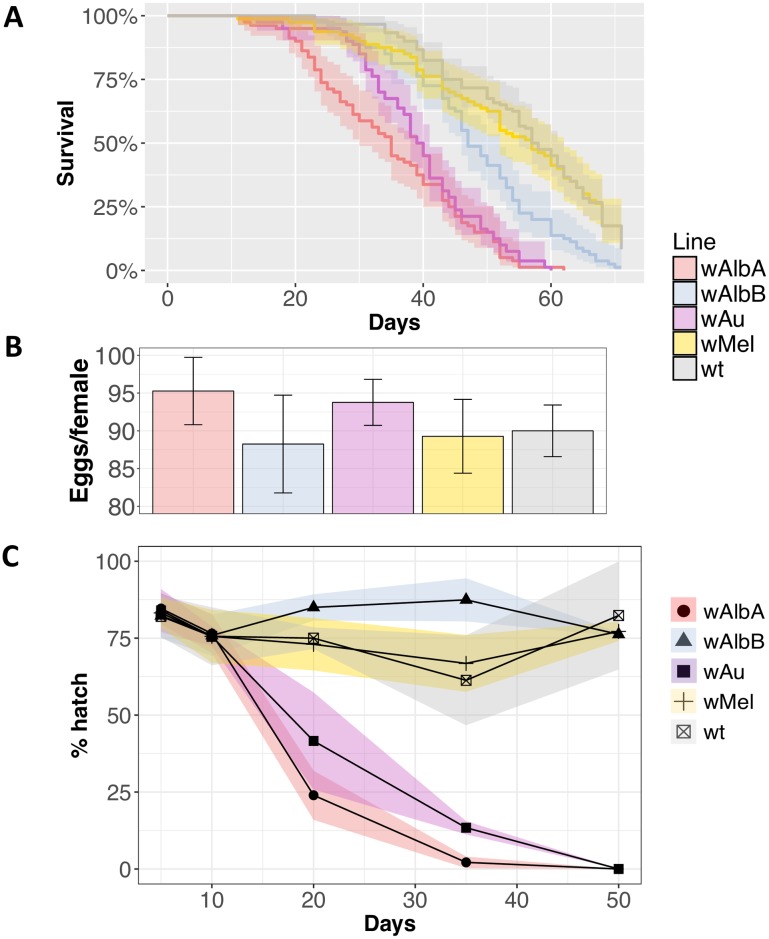
Fitness assessment of *Wolbachia*-infected and wild-type *Ae*. *aegypti*. (**A**) Survival of adult females of *Wolbachia*-infected lines. Curves show percentage survival with shaded areas indicating 95% confidence intervals from 4 replicate cages for each line each containing a starting number of 25 adult females. (**B**) Fecundity of females from *Wolbachia*-infected lines and wild-type over the first gonotrophic cycle. 20 females were individualized for oviposition. Bars show average egg number per female. Error bars show SD. (**C**) Percentage hatch rates of eggs from *Wolbachia*-infected lines and wild-type mosquitoes after 5, 10, 20, 35 and 50 days of desiccated quiescence. For each time-point the number of eggs assessed varied from 200–500. Shaded areas around lines indicate 95% confidence intervals.

Another important factor when assessing the comparative utility of *Wolbachia* strains for disease control is their effect on host fitness [37]. The dynamics of cytoplasmic incompatibility, where reproductive advantage afforded to *Wolbachia*-infected females is frequency-dependent, dictate that invasion cannot occur until *Wolbachia* frequency has exceeded a threshold level, and this threshold is in part determined by the effects of the bacteria on host fitness. We assessed important fitness parameters previously shown to be influenced by *Wolbachia* infection: longevity (Fig 4*A*), fecundity (Fig 4*B*) and egg hatch following a period of desiccated quiescence (Fig 4*C*).

Out of all the lines tested, *w*Mel was the only *Wolbachia* strain that did not cause a significant reduction in adult female longevity compared to uninfected wild-type in these laboratory-cage experiments (*p*>0.1). *w*AlbB caused a slight but significant reduction in the longevity of females. The *w*Au and *w*AlbA infected lines resulted in the most significant reductions in female longevity, consistent with the hypothesis that the higher density *Wolbachia* strains cause the highest fitness costs (*p*<0.001). Similar trends in longevity were observed in males (S2 Fig).

No influence of any of the *Wolbachia* strains on female fecundity was detected when compared to wild-type hatch rates (*p* = 0.91, 1-way ANOVA with Dunnett’s) (Fig 4*B*), with all strains producing approximately 90 eggs per female on average. A strain-dependent effect on the ability of eggs to survive in desiccated quiescence was observed. The wild-type, *w*Mel and *w*AlbB lines showed no significant reductions in egg hatch rates after 50-days of quiescence compared with their respective 5-day hatch rate (*p*>0.4 for each comparison, t-test). However, the *w*Au and *w*AlbA containing lines showed reductions in egg survival with time over this period. The effect was strongest in the *w*AlbA line, with hatch rates dropping to 50% after approximately 13 days of desiccated quiescence. The hatch rate of the *w*Au infected line dropped to 50% after approximately 18 days.

#### A *w*Au—*w*AlbB superinfection causes uni-directional CI

**Fig 5:**
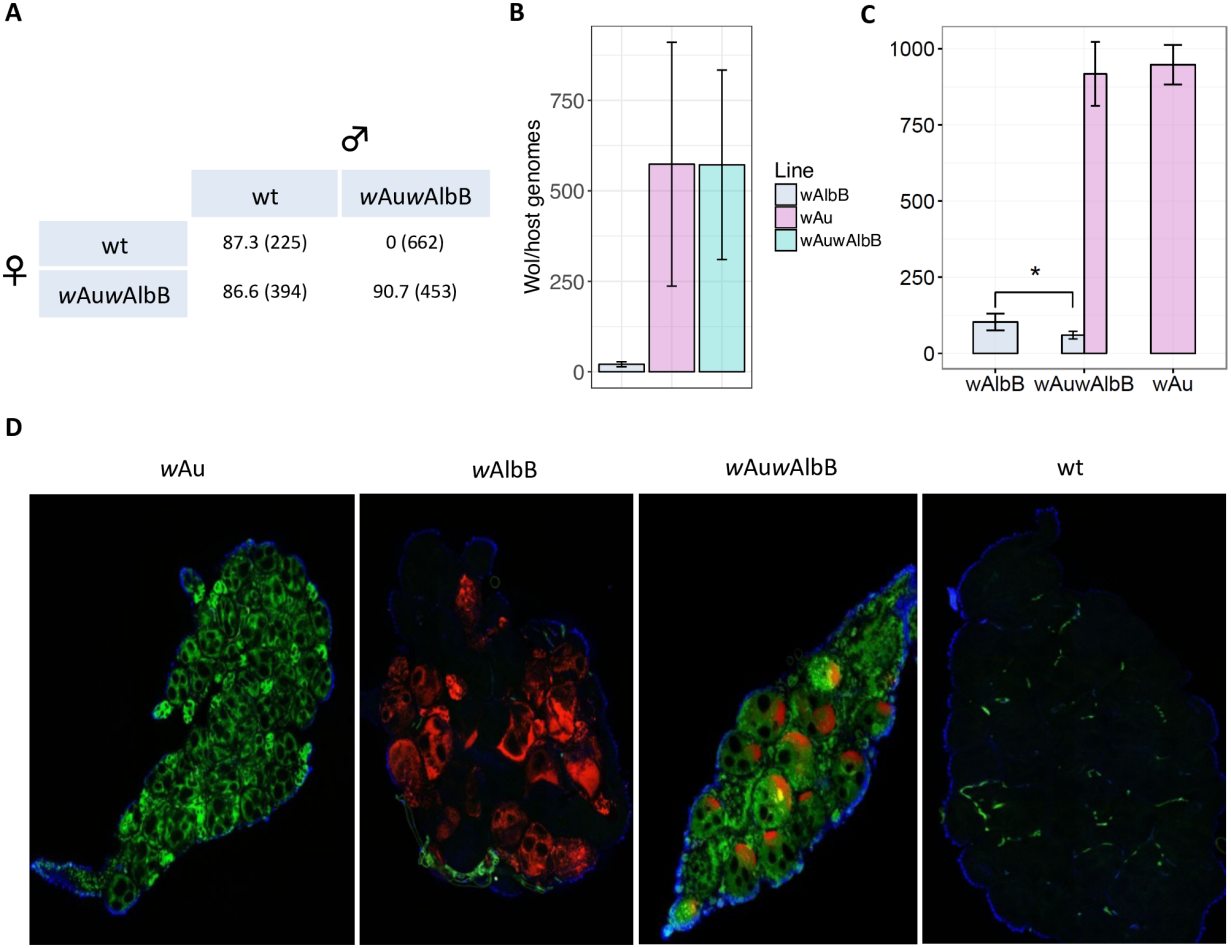
Generation of a *w*Au – *w*AlbB superinfection. (**A**) Crosses between *w*Au*w*AlbB and wild-type lines. Eggs are from crosses of 20 males and 20 females. Numbers show percentage hatch rates with total numbers of eggs counted in parentheses. (**B**) Total *Wolbachia* densities measured by qPCR in *w*AlbB, *w*Au, and *w*Au*w*AlbB carrying *Aedes aegypti* females at ten days post adult eclosion. Each bar represents 10 biological replicates, with pools of 5 females per replicate. Error bars show SD. (**C**) *w*Au and *w*AlbB strain-specific densities in the ovaries of *w*AlbB, *w*Au*w*AlbB, and *w*Au carrying *Ae*. *aegypti*. Each bar represents the average densities from 5 biological replicates each containing ovaries of 10 adult females. Error bars show SD. Statistical analysis was performed using a one-way ANOVA. (**D**) Fluorescent *in situ* hybridization showing distributions of *w*Au (green) and *w*AlbB (red) in ovaries of the *w*Au, *w*AlbB, *w*Au*w*AlbB and wild-type (wt) lines. For all images ovaries were treated with both red and green probes. A no-probe control showing some green auto-fluorescence in wild-type ovaries is shown in S4 Fig. Blue stain is DAPI.

The *w*Au strain was able to successfully establish itself in wild populations of *Drosophila simulans* without inducing CI [36]. Given that densities and associated fitness costs in *Ae*. *aegypti* are likely to be lower in wild mosquitoes than in the lab, and that its maternal transmission fidelity is very high, it is possible that *w*Au could maintain itself in field *Ae*. *aegypti* following introduction. Alternatively, it could be driven into an uninfected population by combining *w*Au with a *Wolbachia* strain capable of causing unidirectional CI. As a proof-of-concept, we created a *w*Au superinfection using the *w*AlbB strain as the ‘driver’, since *w*AlbB combines unidirectional CI with temperature stability and relatively strong viral inhibition. A superinfected line was generated by transferring cytoplasm from *w*Au-carrying *Ae*. *aegypti* embryos to embryos carrying *w*AlbB. As expected, the *w*Au*w*AlbB line produced full unidirectional CI when crossed with wild-type mosquitoes (Fig 5*A*). Analysis of adult females showed that the *w*Au*w*AlbB line possesses very similar over-all densities to the *w*Au single-infection (Fig 5*B*). Moreover, a comparison of the *w*Au, *w*AlbB and *w*Au*w*AlbB lines revealed that the presence of *w*AlbB did not significantly reduce the density of *w*Au in *w*Au*w*AlbB ovaries (Fig 5*C*) (*p*>0.1, t-test), suggesting that *w*AlbB will not affect the maternal transmission rate of *w*Au in the superinfected line, and no reductions in *w*Au density were found in the midgut or salivary gland tissues of the *w*Au*w*AlbB line compared to the *w*Au-only line (S3 Fig), strongly suggesting that *w*Au*w*AlbB will display a similar virus blocking and fitness profile to the *w*Au-only line. However, *w*AlbB ovary density was significantly reduced in the presence of *w*Au (Fig 5*C*) (*p*<0.03, t-test), although this does not appear to impact the capacity of *w*AlbB to rescue CI (Fig 5*A*). The infections in the *w*Au and *w*AlbB single and superinfected lines were visualized by whole-mount fluorescence *in situ* hybridization, using separate *w*Au (green) and *w*AlbB-specific (red) probes (Fig 5*D*). The images obtained show that *w*Au is present in a greater number of ovarian cells and occupies a greater volume within the cells compared with *w*AlbB, which is notably more restricted in its distribution.

## Discussion

In light of the failure to establish *w*MelPop in wild populations [24], and with the finding that *w*Mel densities are unstable under high temperature treatments [25, 26], it is important to investigate the properties of additional *Wolbachia* strains in *Ae*. *aegypti*. The novel lines generated here highlight the variability in phenotypic effects caused by different *Wolbachia* strains in a common host background, and emphasizes the difficulties in making reliable predictions of phenotype based solely on observations of the strain in single host species.

The high level of virus inhibition by *w*Au observed here is consistent with results obtained in *Drosophila* species. A comprehensive assessment of *Wolbachia*-mediated antiviral protection, comparing *Drosophila* C virus (DCV) and Flock House virus (FHV) inhibition by 19 *Wolbachia* strains in same background of *D*. *simulans*, found that *w*Au caused the strongest blocking of both viruses, greater than both *w*Mel and a higher density *w*Mel variant, *w*MelCS [38]. Similar observations of stronger blocking of DCV and FHV by *w*Au relative to *w*MelCS have also been made in *D*. *melanogaster* [34]. Although *w*Au produces some costs to host fitness, these were modest compared to the *w*MelPop strain. In previous experiments carried out by McMeniman and colleagues [23], the median longevity of *w*MelPop-infected females was found to be approximately 26 days, compared to 62 days for wild-type controls. The wAu line produced median female longevity of 40 days, compared to 60 days for wild-type controls.

It was surprising to find significantly higher densities of *w*AlbA than *w*AlbB in *Ae*. *aegypti*, given that *w*AlbA is maintained at much lower densities than *w*AlbB in its native host *Ae*. *albopictus* [31], and is strongly suggestive of the presence of host factors/interactions determining *Wolbachia* density in a strain-specific fashion, rather than simple differences in replication rates between *Wolbachia* strains. The influence of host factors has been previously suggested, when densities of *w*MelPop were found to vary significantly between the native host *D*. *melanogaster* and the closely related *Drosophila simulans* [39]. Studies comparing *w*Mel with the high-density variant *w*MelPop have correlated duplications of a region of eight genes with increases in *Wolbachia* density in the native host *Drosophila melanogaster* [27, 29, 30]. However, this region is completely deleted in other *w*MelPop sub-strains [40]. Moreover, *w*Au lacks this locus [34], but reaches higher densities than *w*Mel and provides greater pathogen protection in *D*. *simulans* [35]. The apparent strain-specific nature of *Wolbachia* density control is encouraging in terms of maximizing the long-term effectiveness of *Wolbachia*-based strategies for virus control. Even if mean *Wolbachia* density reduction occurs over time due to selection on the host, and thus amelioration of virus transmission-blocking, other strains could subsequently be introduced to restore high density and thus the effectiveness of disease control.

We show that the effects of high temperature on density can vary dramatically between *Wolbachia* strains, and confirm previous studies showing that *w*Mel is particularly susceptible to maternal leakage over consecutive generations of heating. The upper temperature used here is high but realistic for larvae in tropical regions [41]. Choosing *Wolbachia* strains that show the greatest density stability of natural environments where releases take place should therefore be a key concern when considering the suitability of strains in a given location. Higher temperature conditions may result in lower *Wolbachia* densities in the field, which could cause reduced pathogen inhibition. However lower densities also correlate with lower fitness costs; high temperatures may therefore also result in improved fitness characteristics and population spread capacity. Likewise, in hot tropical regions without a marked dry season, reduced embryo hatch after quiescence may have little impact on spread dynamics. The direct comparison of *Wolbachia* strains presented here also highlights the utility of *w*AlbB, which combines similar levels of virus blocking to *w*Mel, with greater temperature stability—suggesting it may be more effective at spreading and blocking virus transmission in very hot climates.

The demonstration of a stable superinfected line carrying *w*Au and *w*AlbB demonstrates one of several possible methods by which *w*Au could be spread through populations. When used in combination with a ‘driver’ strain, there is always the risk that a decoupling of the strains may occur over time in the field, although the rate at which this would occur is difficult to predict, and may vary between environmental conditions and thus locations. Further experiments can explore different strain combinations with *w*Au to maximize co-transmission stability under field-approximating conditions, but the driver strain should also reduce or block virus transmission in case *w*Au is lost, as is the case for *w*AlbB. The combination of two *Wolbachia* strains was previously reported in *Ae*. *aegypti*, where *w*AlbB was stably combined with *w*Mel, resulting in a superinfected strain that showed unidirectional CI with wt, *w*AlbB-only and *w*Mel-only lines [12]. Interestingly the superinfected line showed increased levels of pathogen inhibition compared to the constituent strains.

The recent discovery in *w*Mel that at least two of the genes required for CI induction form an operon located in an integrated WO prophage region [42, 43], which is notably absent in the *w*Au genome [44], opens the intriguing possibility that *w*Au could be converted into a CI-inducing stain following integration of a suitable WO phage element. Crossing-type conversion has been previously reported in *Nasonia* wasps, whereby an incompatibility phenotype was transferred between different strains following innoculation with a 0.23μm pore-filtred pupal homogenate [45]. Overall fitness benefits are also possible under some field conditions, perhaps including protection from harmful viruses, as hypothesized for *w*Au in its native host *Drosophila simulans—*where it is capable of spreading and maintaining high population infection frequencies [36]. Little is known about the frequency of natural entomopathogens to which *w*Au could provide protection in *Ae*. *aegypti*. *w*Au could also potentially be spread through a mosquito population by applying suitable selection pressures such as bacterial, fungal or viral entomopathogens; *Wolbachia w*MelPop has for example been shown to provides resistance to several such agents [46]. In addion to the applied potential of *w*Au, the differences in virus inhbiiton between *w*Au and *w*AlbA, despite reaching similar densities in *Ae*. *aegypti*, provide excellent *in vivo* systems for comparative studies to better understand the mechanistic basis of the phenotype.

## Methods

### Mosquito strains and rearing

The *Ae*. *aegypti* wild-type line used was colonized from Selangor State, Malaysia in the 1960s. All mosquito colonies were maintained at 27°C and 70% relative humidity with a 12-hour light/dark cycle. Larvae were fed tropical fish pellets (Tetramin, Tetra, Melle, Germany) and adults were given access to a sucrose meal *ad libitum*. Blood meals were provided using a Hemotek artificial blood-feeding system (Hemotek, UK) using defribrinated sheep blood (TCS Biosciences, UK). Eggs were collected by providing damp filter-paper (Grade 1 filter paper, Whatman plc, GE healthcare, UK) for oviposition. Eggs were desiccated for 5–10 days prior to hatching in water containing 1g/L bovine liver powder (MP Biomedicals, Santa Ana, California, USA).

### Generation of *Wolbachia*-infected lines

*w*Mel, *w*AlbA and *w*AlbB *Ae*. *aegypti* lines were generated by transferring cytoplasm from superinfected *Ae*. *albopictus* (origin Indonesia) embryos carrying *w*Mel, *w*AlbA and *w*AlbB to wild-type *Ae*. *aegypti* embryos. Microinjections were performed using methods described previously [17]. Female G_0_ survivors were back-crossed to wild-type males, blood-fed and separated individually for oviposition. G_0_ females were analysed for *Wolbachia* infection by strain specific PCR (see primer table in Supporting Information for sequences) and eggs from *Wolbachia* negative G_0_ females were discarded. Eggs of positive females were hatched and G_1_’s were assessed for *Wolbachia* G_0_-G_1_ germ-line transmission. Injections from the superinfected *Ae*. *albopictus* line initially resulted in the generation of a triple-infected *Ae*. *aegypti* line (*w*Mel*w*AlbA*w*AlbB), which showed unstable maternal inheritance of *Wolbachia* strains. Individualizing the progeny of triple infected females resulted in the isolation and establishment of the *w*AlbA-only, *w*AlbB-only and *w*Mel-only lines. The *w*Au line was generated as above, but involved transfer of cytoplasm from *w*Au infected *Drosophila simulans* embryos (origin Australia). The *w*Au*w*AlbB line was generated as above but involved the transfer of cytoplasm from the *w*Au-infected *Ae*. *aegypti* line into embryos of the *w*AlbB-infected line.

### Maternal inheritance and CI

To assess rates of maternal inheritance, females from each *Wolbachia* transinfected line were crossed to wild-type males in pools of 20 males and 20 females. A blood-meal was provided and females were individualised for oviposition. The resulting eggs were hatched and DNA from a selection of 10 of these (200 assessed for each line in total) was extracted at the pupal stage and a PCR for *Wolbachia* was performed.

Rates of CI induction and rescue both with wild-type mosquitoes and between infected lines were assessed by crossing 20 males and 20 females of each line. A blood-meal was provided and females were individualised for oviposition. Eggs were collected on damp filter paper, which was subsequently desiccated for 5 days at 27°C and 70% relative humidity. Eggs were counted and hatched in water containing 1g/L bovine liver powder. Larvae were counted at the L2-L3 stage to provide hatch rates. Females with no egg hatch were dissected to check spermathecae for successful mating. Unmated females were excluded from hatch rate evaluations.

### *Wolbachia* strain-specific PCR and density qPCR

For PCR analysis, genomic DNA was extracted from mosquitoes using the Livak method [47]. For primer sequences see primer table in supporting information. For measurements of *Wolbachia* density by qPCR, genomic DNA was extracted from mosquitoes using phenol/chloroform. Unless stated otherwise, mosquitoes used in density experiments were adults 5-days post pupal eclosion. gDNA was diluted to 100ng/μl using a NanoDrop spectrophotometer (Thermo Scientific, Waltham, Massachusetts, USA). A BioRad CFX-96 real-time PCR detection system was used (Bio Rad, Hercules, California, USA) with 2 x SYBR-Green mastermix (Biotool, Houston, Texas, USA). Total *Wolbachia* density was analysed by absolute quantification against a dilution curve of a vector containing single copies of the homothorax (HTH) gene and *Wolbachia* surface protein (*wsp*).

To specifically quantify the *w*AlbA, *w*AlbB, *w*Au, and *w*Mel strains, the following primers were used: *w*AlbA–(QAdir1 and QArev2); *w*AlbB–(183F and QBrev2); *w*Au–(*w*AuF and *w*AuR); wMel–(qMel-F and qMel-R). All were normalized against HTH copies. The following program was used to run the qPCRs: 95°C for 5mins, 40x cycles of 95°C for 15sec and 60°C for 30sec, followed by a melt-curve analysis. Primer sequences can be found in S1 Table.

### Fluorescent *in situ* hybridization

Ovaries were dissected from 5-day old adult females in a drop of PBS buffer, and were immediately transferred to a tube containing Carnoy’s fixative (chloroform:ethanol:acetic acid, 6:3:1) and fixed at 4°C overnight. Samples were then rinsed in PBS and transferred to a 6% hydrogen peroxide in ethanol solution for 72 hours at 4°C. Samples were then incubated in a hybridization solution containing: 50% formamide, 25% 20xSSC, 0.2% (w/v) Dextran Sulphate, 2.5% Herring Sperm DNA, 1% (w/v) tRNA, 0.015% (w/v) DTT, 1% Denhardt’s solution, and 100ng/ml of each probe. Probe sequences were as follows: *w*Au (green) 5’-ACCTGTGTGAAACCCGGACGAAC-(Alexa flour 488)-3’; *w*AlbB (Red) 5’-TAGGCTTGCGCACCTTGCAGC-(Cyanine3)-3’. Samples were left to hybridize overnight in a dark-damp box at 37°C. Samples were washed twice in a solution containing: 5% 20xSSC, 0.015% (w/v) DTT, and twice in a solution of 2.5% SSC, 0.015% (w/v) DTT in dH2O, with each wash performed at 55°C for 20 minutes. Samples were then placed on a slide containing a drop of VECTASHIELD Antifade Mounting Medium with DAPI (Vector Laboratories, California, USA) and were visualized immediately using a Zeiss LSM 880 confocal microscope (Zeiss, Oberkochen, Germany). Both the red and green probes were added to the hybridization solution to produce the images of *w*AlbB, *w*Au, *w*Au*w*AlbB and wild-type ovaries.

### Thoracic injection of SFV

Twenty 5-day old female mosquitoes of each *Wolbachia*-infected line and wild-type were injected with the respective virus in the thorax using a pulled glass capillary and a Nanoject II (Drummond Scientific, Pennsylvania, USA) hand-held microinjector. Injected mosquitoes were immediately transferred to an incubator set at 27°C and a 12-hour light/dark cycle for recovery. SFV injected females were left for ten days prior to RNA extraction and virus quantification by qRT-PCR. RNA was extracted using TRI Reagent (Sigma-Aldrich, Missouri, USA). cDNA was synthesized using 1μg of total RNA and the All-In-One cDNA Synthesis SuperMix (Biotool, Houston, Texas, USA). qRT-PCRs were performed on a 1 to 20 dilution of the cDNAs. Virus levels were normalized to the RPS17 house-keeping gene.

Semliki Forest virus was sub-type C (catalogue number 1112041v) obtained from Public Health England culture collections. SFV was propagated on C6/36 cells to a final injection concentration of 1.78x10^13^ FFU/ml. Primers used for viral detection were: SFV4-F and SFV4-R.

### Oral infections with ZIKV and DENV

Five day-old females were fed an infectious blood-meal containing a mixture of 800μl defibrinated sheep blood and 400μl viral suspension supplemented with a phagostimulant (ATP) at a final concentration of 5mM. Dengue virus was serotype 2, New Guinea C strain, obtained from Public Health England culture collections. Zika virus was strain MP1751, obtained from Public Health England culture collections. The final concentration of dengue virus in the blood meal was 8.3x10^7^ FFU/ml. The final concentration of Zika virus in the blood meal was 1.6x10^8^ FFU/ml. Engorged females were separated and maintained in a climactic chamber at 27°C and 75% humidity. After 12 days females were salivated by inserting the proboscis into a capillary containing mineral oil and placing a drop of 1% pilocarpine nitrate onto the thorax. Collected saliva was ejected into tubes containing Dulbecco’s Modified Eagle Medium (DMEM) medium supplemented with 2% fetal bovine serum (FBS), 10-fold serially diluted, and added to pre-seeded Vero cells for fluorescent focus assay (FFA). Primary antibody for dengue was the MAB8705 Anti-Dengue Virus Complex Antibody clone D3-2H2-9-21 (Millipore, Massachusetts, USA). Primary antibody for Zika was the MAB10216 Anti-Flavivirus Virus Complex Antibody clone D1-4G2-4-15 (Millipore, Massachusetts, USA). Secondary antibody for both viruses was the Goat anti-mouse Alexa Fluor 488, A-11001 (Thermo Scientific, Waltham, Massachusetts, USA). Plates were imaged using a Typhoon 9400 plate reader (GE Healthcare, Little Chalfont, UK) and images were analysed using ImageJ (NIH, USA).

Once saliva was collected, mosquito salivary glands were dissected and RNA was extracted using the QIAamp Viral RNA Mini kit (Qiagen, Hilden, Germany) according to manufacturers guidelines. Abdomens were removed and placed into tubes containing RNAzol reagent (Sigma-Aldrich, Missouri, USA). RNA was extracted according to manufacturers guidelines. cDNA synthesis was performed using the All-In-One cDNA Synthesis SuperMix (Biotool, Houston, Texas, USA), and qPCRs were run using NS5-F and NS5-R primer set for dengue and the ZIKV 835 and ZIKV 911c primers for Zika virus. For Zika infected mosquitoes, the numbers of samples analysed were 22, 21, 16 and 18 for *w*AlbB, *w*Au, *w*Mel and wild-type, respectively. For dengue infected mosquitoes the numbers of samples analysed were 19, 18, 19 and 17 for *w*AlbB, *w*Au, *w*Mel and wild-type, respectively. Levels of target cDNA sequences were normalized against the RpS17 house-keeping gene using the Pfaffl method. Primer sequences can be found in S1 Table.

### *Wolbachia* response to temperature fluctuations

Eggs of the *w*AlbA, *w*AlbB, *w*Au and *w*Mel strains were hatched under either constant 27°C (control) or 27–37°C at a 12:12hr cycle (heat-stressed) in a Panasonic MLR-352-H Plant Growth Chamber incubator (Panasonic, Osaka, Japan) and corresponding light and dark photoperiods (light during 37°C). Immediately upon hatching, larvae were picked and placed into trays containing 1L of water and larval food at a density of 50 larvae per tray (three replicate trays per strain). Larvae were reared until pupation with water temperatures monitored daily using a glass thermometer placed inside a water-filled beaker. Water in the larval trays was replaced every 2 days to reduce bacterial growth. A selection of adults was removed upon emergence and split into two groups with a batch of approximately 15 (pooled into 5 repeats each containing 3 pooled adults) analysed by qPCR for *Wolbachia* density using the WSP and HTH primer sets. The remaining adults were set up in cages maintained at a constant 27°C and allowed to recover for 7 days. These were also split into two groups with a sub-set analysed by qPCR for *Wolbachia* density, and the remainder (five females from each line) mated to wild-type males and blood-fed at day 5-post emergence. Eggs were collected and hatched at constant 27°C and reared to pupation. A selection of 10 pupae from each female were chosen at random and assessed for *Wolbachia* infection by PCR.

A subset of adult females emerging from heat-stressed larvae were maintained under temperature cycling, mated to wild-type males, blood fed at day 5 post emergence and allowed to oviposit. Eggs were hatched and reared to pupation under heat stress at which point a selection of 10 pupae from each female were chosen at random and assessed for *Wolbachia* infection by PCR.

### Adult longevity

Adult survival was assessed using groups of 50 individuals at a sex ratio of 1:1, with four replicates for each line. Experiments were performed in 24.5x24.5x24.5cm insect rearing cages inside an incubator set to 27°C and 70% relative humidity with a 12-hour light/dark cycle. Cages were blood-fed once a week from day 5 onwards and damp filter paper was provided for oviposition. A sucrose meal was accessible *ad libitum*. Cages were checked daily for mortality. Experiments ran for 70 days at which time approximately 10% of the *w*Mel and wild-type females remained alive.

### Fecundity

Female fecundity was assessed by feeding 5-day old males and females of *Wolbachia*-infected and wild-type mosquitoes on a hemotek feeder containing defribrinated sheep blood. 20 fully engorged females (considered fully engorged when a female had a full abdomen and voluntarily dropped off the blood source) were isolated using an aspirator. Females were placed individually inside up-turned cups on top of a circle of filter paper. Cotton-wool soaked in a 10% sucrose solution was made available through a hole in the cup. 3 days post-feeding the filter paper was wetted and left overnight. The filter paper was replaced the next day and the process was repeated for a second night. Eggs from each filter paper were counted using a clicker-counter and a dissecting microscope.

### Egg survival

Egg survival in desiccated quiescence was assessed by feeding one week old *Wolbachia*-infected or wild-type females in cages and collecting eggs 3 and 4 days after feeding by placing three separate damp filter-paper cones in each cage—each cone collected >1,000 eggs. Egg papers were stored at 27°C and 70% relative humidity. At 5, 10, 20, 35 and 50-days post oviposition a section of each of the egg papers containing approximately 200–300 eggs was cut from the original paper, the eggs counted using a clicker-counter and dissection microscope, and hatched by placing in water containing 1g/L bovine liver powder. Hatch rates were assessed 10 days later by counting larvae using a Pasteur pipette and a clicker-counter.

### Statistical analysis

All statistical analyses were performed in the RStudio interface (version 0.99.489) (RStudio Inc., Boston, Massachusetts, USA) of the R software (version 3.4.0). Graphics were generated using the ‘ggplot2’ package. Normality of data distributions were assessed using a Kolmogorow-Smirnov Test prior to hypothesis test selection. Multiple comparisons were performed using the ‘multcomp’ package and used the Bonferroni multiple comparisons *p* value correction method. Survival analyses were performed using the ‘Survival’ and ‘SurvMiner’ packages. Survival analyses were performed using a Cox proportional hazard regression model with cage repeats clustered as a random effect.

#### Primer sequences

A list of primers and primer sequences used in this manuscript can be found in supplementary Table 1.

## Supporting information

S1 Fig Zika (ZIKV) virus genome copies per host cell in salivary gland tissues following oral infection. Zika virus was orally administered to 5-day old females. After an incubation period of 12 days salivary glands were dissected. Viral RNA was quantified by reverse-transcriptase qPCR, with viral RNA levels normalized to host RNA using the *RpS17* house-keeping gene.
(TIF)

S2 Fig Longevity of male *Wolbachia*-infected and wild-type *Ae*. *aegypti*. Survival of adult males of *Wolbachia*-infected lines compared to wild-type. Curves show percentage survival with shaded areas indicating 95% confidence intervals from 4 replicate cages for each line each containing a starting number of 25 adult males.
(TIF)

S3 Fig Densities of *w*Au and *w*AlbB in somatic tissues. *w*Au and *w*AlbB strain-specific densities in the midguts and salivary glands of *w*AlbB, *w*Au*w*AlbB, and *w*Au carrying *Ae*. *aegypti*. Each bar represents the average densities from 5 biological replicates each containing ovaries of 10 adult females. Error bars show SD.
(TIF)

S4 Fig No probe FISH control of wild-type ovaries. Fluorescent *in situ* hybridization image of wild-type ovaries taken at the same time as those shown in Fig 5, but hybridization buffer lacked FISH probes. Some green auto-fluorescence is visible.
(TIF)

S1 Table Primer sequences. Sequences of DNA oligonucleotides used for assays described in this manuscript.
(DOCX)
