# Supplementary figures and images for: The Wolbachia strain wAu provides highly efficient virus transmission blocking in Aedes aegypti
Source: PLoS Pathog. 2018 Jan 25;14(1):e1006815. doi: 10.1371/journal.ppat.1006815 (PMC5784998; doi:10.1371/journal.ppat.1006815)

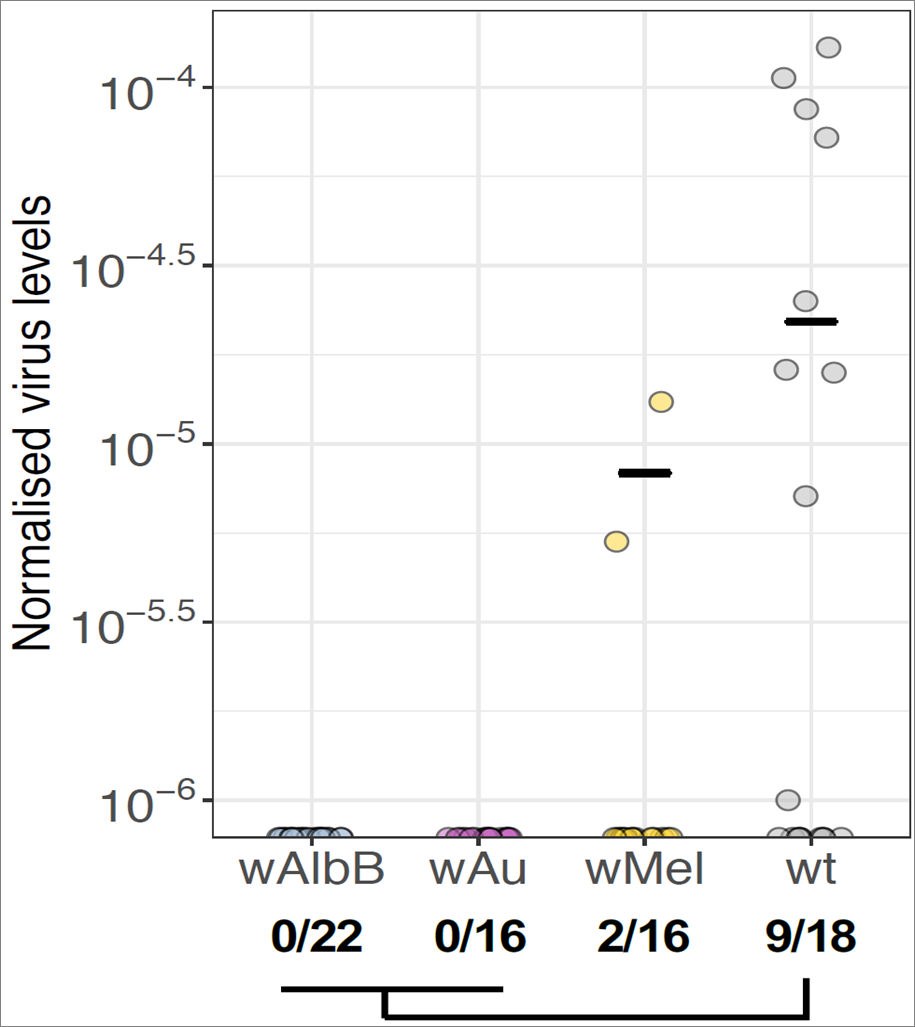

Supplement: S1 Fig — Zika virus was orally administered to 5-day old females. After an incubation period of 12 days salivary glands were dissected. Viral RNA was quantified by reverse-transcriptase qPCR, with viral RNA levels normalized to host RNA using the RpS17 house-keeping gene. (TIF) [file ppat.1006815.s001.tif]

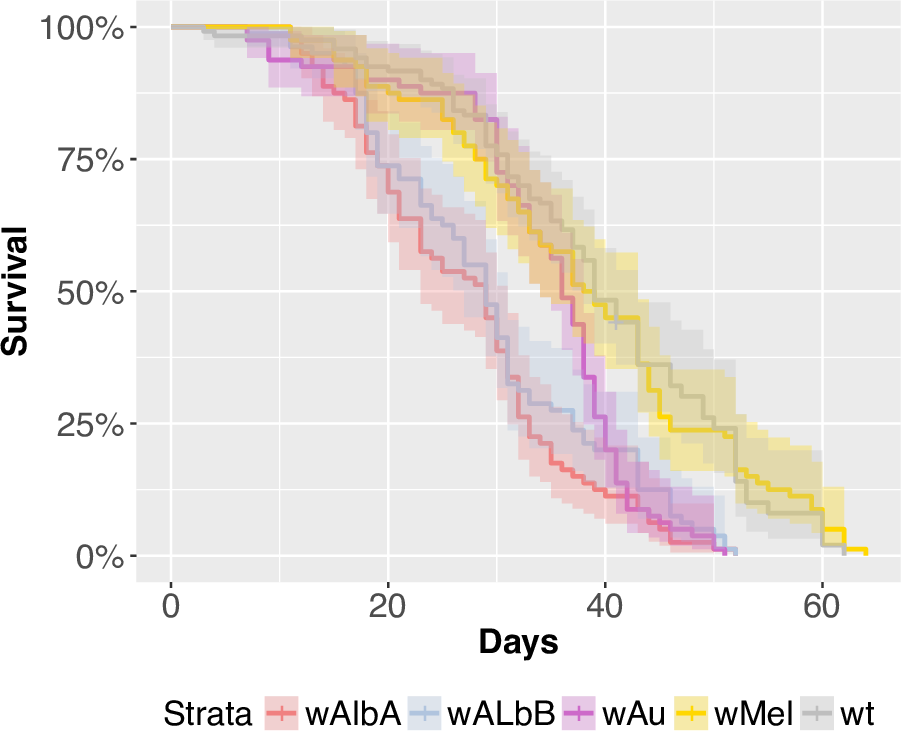

Supplement: S2 Fig — Survival of adult males of Wolbachia-infected lines compared to wild-type. Curves show percentage survival with shaded areas indicating 95% confidence intervals from 4 replicate cages for each line each containing a starting number of 25 adult males. (TIF) [file ppat.1006815.s002.tif]

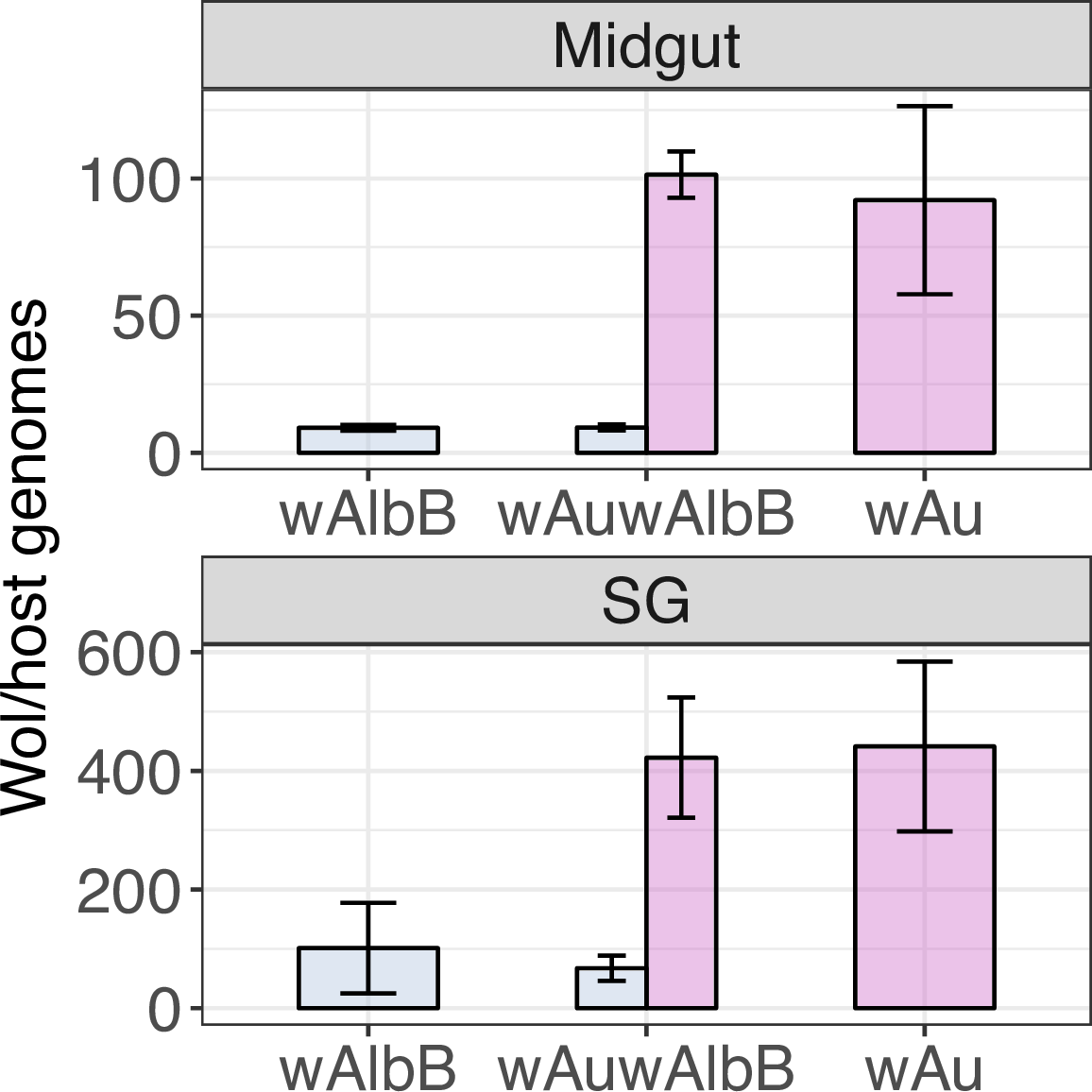

Supplement: S3 Fig — wAu and wAlbB strain-specific densities in the midguts and salivary glands of wAlbB, wAuwAlbB, and wAu carrying Ae. aegypti. Each bar represents the average densities from 5 biological replicates each containing ovaries of 10 adult females. Error bars show SD. (TIF) [file ppat.1006815.s003.tif]

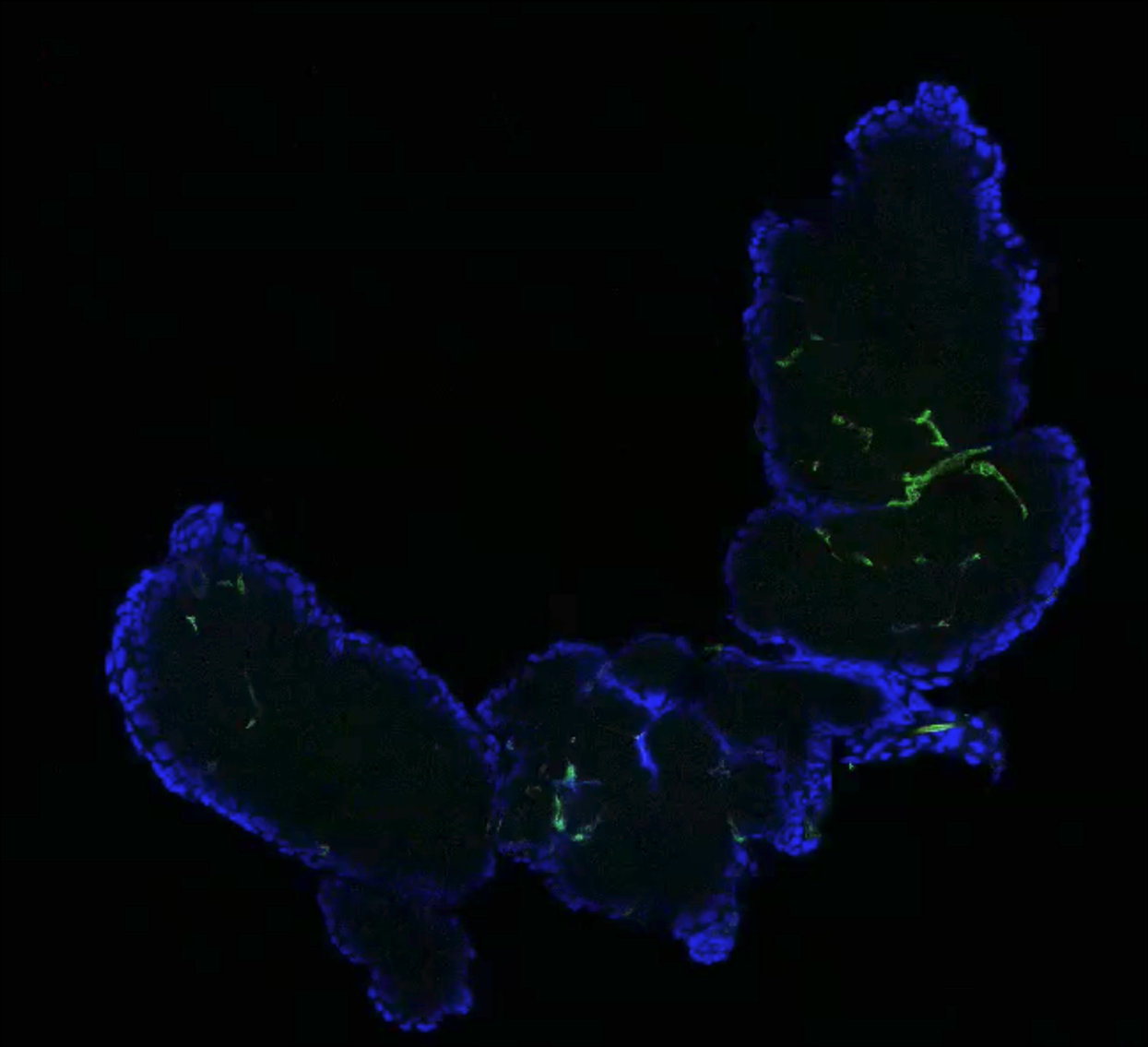

Supplement: S4 Fig — Fluorescent in situ hybridization image of wild-type ovaries taken at the same time as those shown in Fig 5, but hybridization buffer lacked FISH probes. Some green auto-fluorescence is visible. (TIF) [file ppat.1006815.s004.tif]
