# Supplementary material for: The Wolbachia strain wAu provides highly efficient virus transmission blocking in Aedes aegypti
Source: PLoS Pathog. 2018 Jan 25;14(1):e1006815. doi: 10.1371/journal.ppat.1006815 (PMC5784998; doi:10.1371/journal.ppat.1006815)
Supplement: S1 Table — Sequences of DNA oligonucleotides used for assays described in this manuscript. (DOCX) [file ppat.1006815.s005.docx]

**Table S1.**

| Primer name | 5’-3’ Sequence |
| --- | --- |
| *w*AlbA-F | GTAGTATTTACCCCAGCAG |
| *w*AlbA-R | ATCTGCACCAGTAGTTTCG |
| *w*AlbB-F | GCAATACCTATGCCGTTTA |
| *w*AlbB-R | GACGAAGGGGATAGGTTAATATC |
| *w*Mel-F | TATTGAGCCTTCCTCGTACC |
| *w*Mel-R | TAGCATGCCGTTTTTCTGTA |
| *w*Au-F (*wsp*81F) (48) | TGGTCCAATAAGTGATGAAGAAAC |
| *w*Au-R | TTTGCTGGGTCAAATGTTACATCTT |
| qHTH-F (48) | TGGTCCTATATTGGCGAGCTA |
| qHTH-R (48) | TCGTTTTTGCAAGAAGGTCA |
| qWSP-F (6) | ATCTTTTATAGCTGGTGGTGGT |
| qWSP-R (6) | AAAGTCCCTCAACATCAACCC |
| QAdir1 (49) | GGGTTGATGTTGAAGGAG |
| QArev2 (49) | CACCAGCTTTTACTTGACC |
| 183F (50) | AAGGAACCGAAGTTCATG |
| QBrev2 (49) | AGTTGTGAGTAAAGTCCC |
| RpS17-F | CACTCCCAGGTCCGTGGTAT |
| RpS17-R | GGACACTTCCGGCACGTAGT |
| DENV-NS5-F (6) | ACAAGTCGAACAACCTGGTCCAT |
| DENV-NS5-R (6) | GCCGCACCATTGGTCTTCTC |
| SFV4-F (50) | CGCATCACCTTCTTTTGTG |
| SFV4-R (50) | CCAGACCACCCGAGATTT |
| ZIKV-835 (51) | TTGGTCATGATACTGCTGATTGC |
| ZIKV-911c (51) | CCTTCCACAAAGTCCCTATTGC |
| Cecropin-D-F (52) | GCTAGGTCAAACCGAAGCAG |
| Cecropin-D-R (52) | TCCTACAACAACCGGGAGAG |
| Defensin-C-F (6) | TTGTTTGCTTCGTTGCTCTTT |
| Defensin-C-R (6) | ATCTCCTACACCGAACCCACT |
